# High incidence and prevalence of visual problems after acute stroke: An epidemiology study with implications for service delivery

**DOI:** 10.1371/journal.pone.0213035

**Published:** 2019-03-06

**Authors:** Fiona J. Rowe, Lauren R. Hepworth, Claire Howard, Kerry L. Hanna, Christopher P. Cheyne, Jim Currie

**Affiliations:** 1 Department of Health Services Research, University of Liverpool, Liverpool, United Kingdom; 2 Department of Biostatistics, University of Liverpool, Liverpool, United Kingdom; 3 Vision and Stroke Patient and Public Group (VISable), University of Liverpool, Liverpool, United Kingdom; University of Florida, UNITED STATES

## Abstract

**Background:**

Visual problems are an under-reported sequela following stroke. The aim of this study is to report annual incidence and point prevalence of visual problems in an acute adult stroke population and to explore feasibility of early timing of visual assessment.

**Methods and findings:**

Multi-centre acute stroke unit, prospective, epidemiology study (1^st^ July 2014 to 30^th^ June 2015). Orthoptists reviewed all patients with assessment of visual acuity, visual fields, ocular alignment, ocular motility, visual inattention and visual perception. 1033 patients underwent visual screening at a median of 3 days (IQR 2) and full visual assessment at a median of 4 days (IQR 7) after the incident stroke: 52% men, 48% women, mean age 73 years and 87% ischaemic strokes. Excluding pre-existent eye problems, the incidence of new onset visual sequelae was 48% for all stroke admissions and 60% in stroke survivors. Three quarters 752/1033 (73%) had visual problems (point prevalence): 56% with impaired central vision, 40% eye movement abnormalities, 28% visual field loss, 27% visual inattention, 5% visual perceptual disorders. 281/1033 (27%) had normal eye exams.

**Conclusions:**

Incidence and point prevalence of visual problems in acute stroke is alarmingly high, affecting over half the survivors. For most, visual screening and full visual assessment was achieved within about 5 days of stroke onset. Crucial information can thus be provided on visual status and its functional significance to the stroke team, patients and carers, enabling early intervention.

## Introduction

The prevalence of overall visual problems has been estimated at 65% with varying prevalence reported for specific types of visual problems [1–4]. For example, visual field loss is reported in up to 52% of stroke survivors, central visual problems in up to 70%, eye movement disorders in up to 68% and visual perceptual disorders (inclusive of visual inattention) in up to 80% of stroke survivors [1,3,5,6]. The wide ranges relate to the design of source studies with a mix of randomised controlled trials through to observation cohort studies, and also relate to mixed stroke populations, purposeful samples and varying time periods from stroke onset to visual assessment. Figures for incident new onset visual sequelae following stroke are not reported in the published literature.

Accurate information about incidence of visual sequelae and timing of visual assessment is important when considering the needs of stroke survivors and developing services for appropriate and adequate patient care.

### Aims

The aim of this study was to determine the incidence and point prevalence of visual problems in an acute stroke population and to explore the timing at which visual assessment can be first undertaken in this population.

## Methods

### Population

The Impact of Visual Impairment after Stroke (IVIS) study was undertaken in three hospital hyper-acute and acute stroke units in the North West of England. The target population was stroke survivors in the acute phase (within 2 weeks post stroke onset) following admission to hospital with a clinical diagnosis of stroke confirmed by the admitting stroke physician.

Ethical approval was obtained from the Health Regulatory Authority (Research Ethics Committee reference 14/NW/0166) and the study was undertaken in accordance with the Tenets of Helsinki. The research ethics panel waived the need for written patient consent. Thus, written patient consent was not required or obtained for this study. Verbal assent was sought and obtained for all vision assessments in accordance with hospital standard operating procedures. We followed the statement issued by the Association of Medical Research Charities (AMRC) on the use of patient data for research. It is essential to protect confidentiality and for research to do no harm. For our research, we wished to access “anonymised data in which all identifiable information is removed from the data so no link can be made with the individual that it comes from. As these data cannot be linked back to individual people, the data may be shared without seeking further consent“. Using the Health Research Authority ‘No material ethics issue toolkit’ (NMEIT), our study fitted with category 1: Research using data or tissue that is anonymous to the researcher. We followed a capture of assent already in established clinical practice when approaching a patient to offer them vision screening. We used a standard statement all recruitment sites, i.e. “Patient gestured agreement/unable to consent so treatment provided in patient's best interests due to lacking capacity/cognitive impairment/low GCS, etc.”This paper was written in accordance with the STROBE statement [7].

Exclusion criteria were stroke survivors less than 18 years old.

Inclusion criteria were stroke survivors 18 years of age or older with the ability to agree to vision screening using verbal or non-verbal indications of agreement.

### Recruitment

On a daily basis, the stroke research nurse team identified all stroke admissions to each of the three recruiting stroke units. Details of each admitted patient (name, date of birth and hospital identification number) were forwarded to the research orthoptists. First visit for attempted vision screening was made at the next designated orthoptic session on the stroke unit.

Stroke-specialist orthoptist staffing ratios followed national guidance of 1 session (whole time equivalent [WTE]) per 10-beds [8]. Each stroke unit had two orthoptic sessions per week split with one session at the start of the week and one at the end of the week to enable capture of stroke admission across the entire week. In addition, one outpatient orthoptic clinic per hospital was provided solely for stroke survivors follow-up appointments after stroke unit discharge. Thus, each hospital had a total of three orthoptic sessions per week.

### Assessment

Following a review of the hospital notes for previous ocular history and case history taking from the patient and/or carer, a full, new assessment of visual function was made with measurement of:

- visual acuity for near and distance, monocular and binocular (logMAR, Cardiff acuity cards, Vocational near visual acuity),
- reading ability (Radner reading test),
- colour vision (City test) and contrast sensitivity assessment (MARs test),
- ocular alignment assessment (cover/uncover test),
- rotation of eye movements (saccadic and smooth pursuit movements),
- vergence (near point of convergence, divergence ability),
- stereopsis (Frisby test plate),
- fusional vergence (20 prism dioptre base-out, prism fusion range),
- lid and pupil function,
- visual field assessment (visual fields to confrontation, static/kinetic perimetry),
- visual perception (questionnaire),
- visual inattention (line bisection, cancellation task, clock drawing, memory-guided tasks, room description).

All assessments were carried out by stroke specialist orthoptists with expertise in working with this population of patients. Orthoptists are clinicians who specialise in evaluating vision and eye movements working primarily in hospital settings. Assessments on the stroke unit were carried out at the patient’s bedside using portable equipment. Additional vision assessments were conducted where indicated to aid diagnosis. For example, refractive checks were made if glasses history was unclear, or ophthalmic assessments of fundus and media were made to exclude ocular conditions (e.g. cataract, macular degeneration) which had not been previously diagnosed. Assessments were adjusted according to patient needs. For example, patients were allowed to adopt eccentric viewing when testing visual acuity, or allowed to adopt a compensatory head posture to aid binocular viewing. Results of assessments were interpreted accordingly.

Further data were collected with regard to stroke type, gender, age at stroke, ethnicity and stroke severity. Stroke severity was indicated by the Barthel score; a measure of general functional performance based on activities of daily living.

### Categories of visual problems

Types of visual problems were assigned to four categories including:

- Impaired central vision (defined as visual acuity less than 0.3 logMAR equivalent),
- Ocular motility abnormalities (defined as ocular misalignment, incomplete ocular motility [e.g. gaze palsy, cranial nerve palsy, saccadic impairment, smooth pursuit impairment, vergence disorder], impaired binocular vision),
- Visual field loss (defined as loss of part of the central and/or peripheral field of vision, e.g. homonymous hemianopia, quadrantanopia, scotoma),
- Visual perceptual disorders (defined as impaired perception of visual objects or space, e.g. visual inattention, agnosia, alexia).

### Sample and analysis

For this incidence study, we required capture of all stroke admissions over a one-year time period to calculate annual rates. To gather a large cohort, we recruited from three geographically separate hyper-acute and acute stroke units estimated to have approximately 900 stroke admissions per annum.

Descriptive statistics were used to report types of visual problems with categories such as hemianopic visual field loss, ocular motor cranial nerve or gaze palsies and central vision problems. The extent of these types of visual problems were reported numerically as results of visual assessments can be captured as numerical data. For example, area of visual field, logMAR visual acuity measurement and angle of manifest strabismus in prism dioptre measurements are numerical. Independent samples analysis with chi square and Kruskal-Wallis tests were used for evaluation of stroke onset, gender, type of stroke and age. ANOVA was used for parametric comparisons between groups.

### Patient and public involvement

Stroke survivors were involved at the initial set-up of the study and contributed to the development of the study phases. During the first stages of the study, stroke survivors were involved as key stakeholders in focus group meetings. We set up a specific oversight committee, which was chaired and facilitated by stroke survivors. As the study progressed, stroke survivors were actively engaged through to the current dissemination stage.

## Results

During the one-year period of 1^st^ July 2014 to 30^th^ June 2015, there were 1295 stroke admissions across the three hospital stroke units. Results were pooled for analysis for all three stroke units, after an independent samples analysis (chi square test for gender/type of stroke; Kruskal-Wallis test for age/duration) across the three stroke units showed no difference in distribution for gender (*p* = 0.51), age at stroke onset (*p* = 0.15), type of stroke (*p* = 0.51) or duration before full visual assessment was possible (*p* = 0.25).

### General demographics

There were 51.5% males and 48.5% females with a mean age of 73.3 (SD 13.7) years (median of 75 years; range 19–100, Inter Quartile Range (IQR) 19). Stroke type was ischaemic for 87.3% and haemorrhagic in 12.7%. The Barthel score ranged from 0–20 (median 10; mean 9.7, SD 7.8). Ethnicity included white British (94%), white Irish (0.8%), other white (1.6%), Indian (0.7%), Pakistani (0.5%) and Chinese (0.5%). Table 1 outlines demographic data for stroke admissions across the three stroke units and overall.

**Table 1:** Demographics of stroke admissions.

		Site 1	Site 2	Site 3	Total
**Age (years)** **Mean (Standard deviation)**		73.5 (14.1)	72.6 (13.6)	74.1 (13.7)	73.3 (13.7)
**Gender**	**Female**	187	245	196	628
	**Male**	197	243	227	667
**Barthel score** **Mean (Standard deviation)**		9.7 (7.9)	10.5 (7.5)	8.8 (7.8)	9.7 (7.8)
**Stroke type**	**Ischaemic**	332	424	375	1132
	**Haemorrhagic**	52	64	48	163
**Stroke laterality**	**Right**	186	218	189	593
	**Left**	172	254	200	626
	**Bilateral**	26	16	34	76
**Ethnicity**	**White British**	356	459	401	1216
	**White Irish**	6	2	2	10
	**White other**	8	9	4	21
	**Other**	14	18	16	48

### Timing of first visit (baseline) for visual assessment

**Fig 1:**
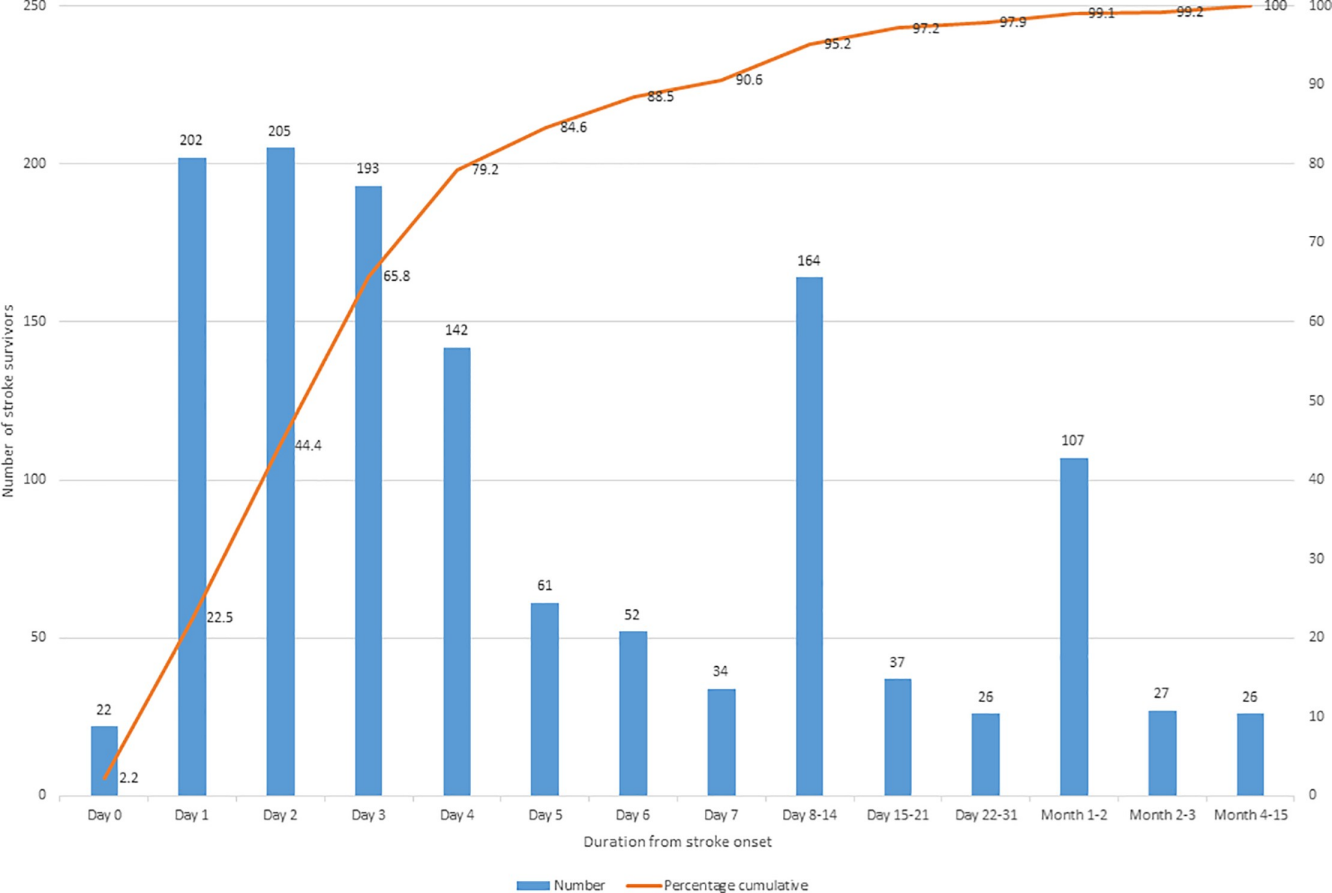
Number of days post stroke onset to first attempted visual assessment. First attempted vision screening was undertaken from day 0 of stroke onset through to a maximum of day 404 (outlier discharged patient who failed to attend earlier outpatient appointments). The majority had been screened within 1 week of stroke onset.

The first visit (baseline) for attempted visual screening assessment was at a mean of 6.5 (SD 24) days (median 3 days; range 0–404, IQR 2). Full visual assessment was undertaken at a mean of 13.4 (SD 33.8) days (median 4 days; range 0–435, IQR 7). On day 0 –the same day as the reported stroke onset– 22 stroke survivors (1.7%) had received a visit for visual assessment; 202 (15.6%) on day 1, 205 (15.8%) on day 2, rising throughout the first week post stroke onset. By the end of one week post stroke onset, 70.5% (n = 911) had received a visit for visual assessment (Fig 1).

### Ability to undertake baseline visual assessment

**Fig 2:**
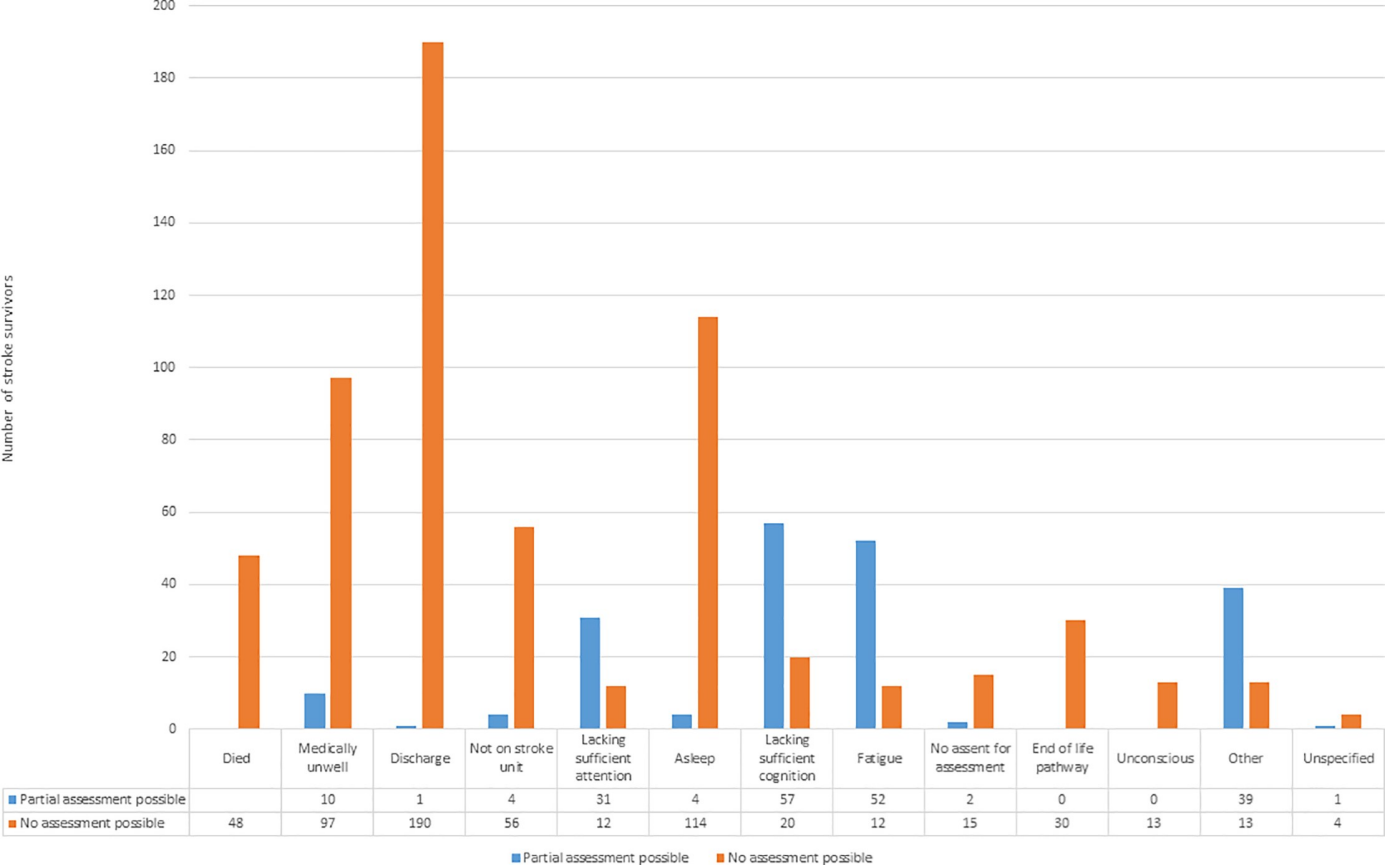
Reasons for lack of visual assessment at visit 0 (baseline). Full vision assessment was not possible at baseline for 627 patients. Reasons for no assessment are outlined in orange. Those with partial, but incomplete, vision assessments are outlined in blue.

Of 1295 stroke admissions at their baseline visit, 668 (51.6%) were able to undergo visual assessment—468 (36.2%) full assessment, 200 (15.4%) partial visual assessment—and 627 (48.4%) could not be assessed at baseline. Reasons for being unable to undergo visual assessment included being medically unwell, lacking sufficient attention or cognition, fatigue and discharge (Fig 2).

**Fig 3:**
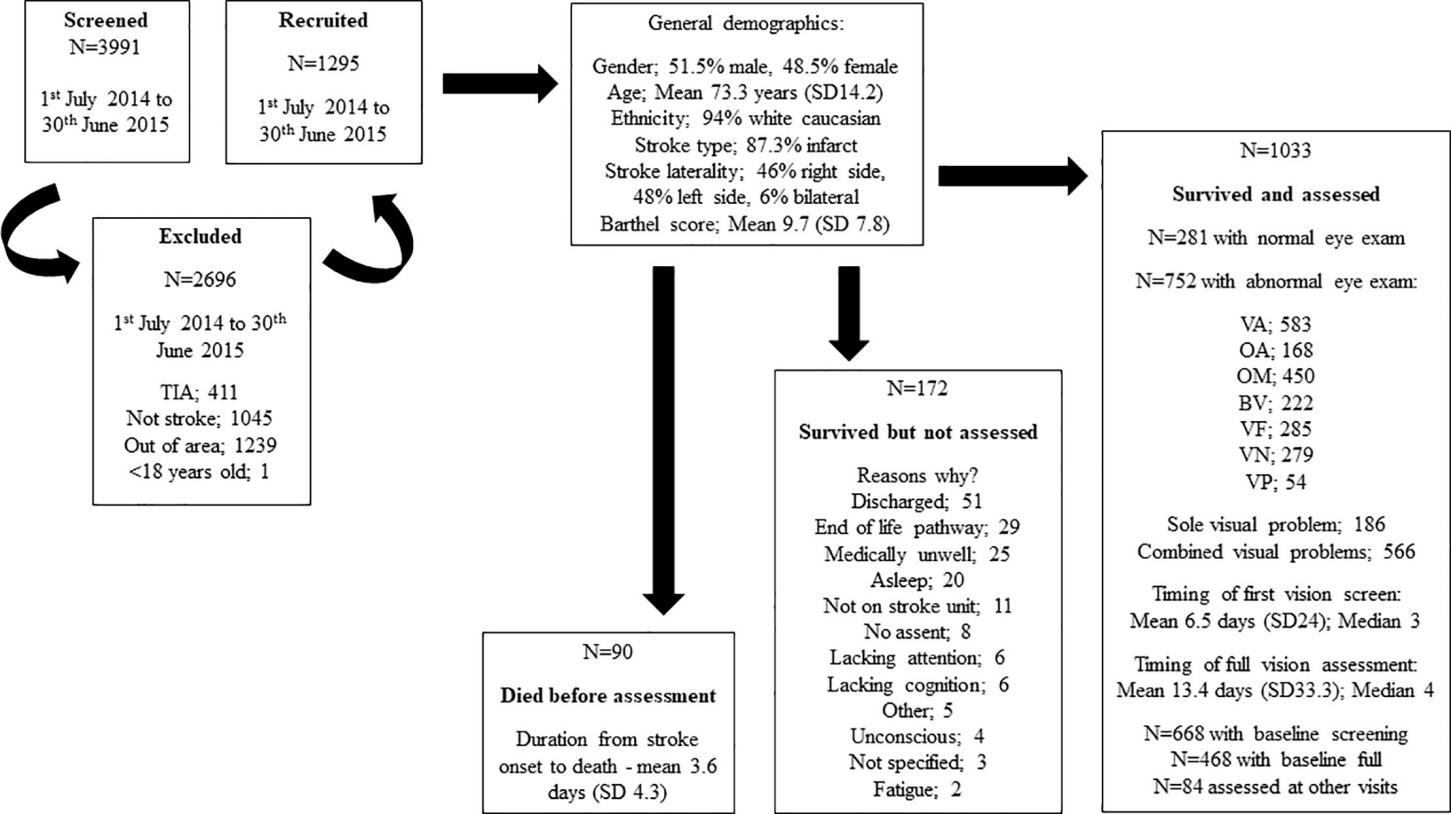
Assessment of post-stroke visual impairment. Breakdown of numbers screened, numbers excluded, number recruited to the study, and numbers of those visually assessed or not.

Of 627 stroke survivors that could not undertake visual assessment at baseline, visual assessment was possible for 365 at subsequent visits (Table 2). The total number of stroke survivors who were never assessed was 262. This included 90 stroke admissions who died prior to any visit for visual assessment with mean duration to death being 3.7 days (SD 3.0). Twenty-six stroke admissions died subsequent to having undergone earlier visual assessment. A further 172 stroke survivors could never be assessed; mainly due to being medically unwell, discharge or being unable to waken the patient for assessment (Fig 3). Those discharged prior to any visual assessment being achieved, were discharged at a mean of 25.8 days (SD 60). Discharge from the stroke unit for all stroke survivors was made at a mean of 38.1 days (SD 64.6).

**Table 2:** Timing from no assessment at baseline to achieving full visual assessment.

Assessment not possible at any visit						172	Died			90
**Visit**	**1**	**2**	**3**	**4**	**5**	**6**	**7**	**8**	**9**	**12**
**n =**	246	68	28	7	7	4	2	1	1	1

### Outcome of visual assessment–point prevalence

Of 1295 stroke admissions, 262 (20.2%) could not undertake a visual assessment and 1033 (79.8%) stroke survivors completed visual assessment. Normal visual assessments were documented for 281 (21.7% of stroke admissions, 27.2% of vision assessments) and 752 (58% of stroke admissions, 72.8% of vision assessments) had abnormal findings on their visual assessments.

Discharge from the stroke unit for stroke survivors with normal visual assessments was made at a mean of 13.5 days (SD 45.9) in comparison to mean discharge of 49.9 days (SD 68.3) for those with visual problems. There was a significant difference in mean discharge periods for those with normal or abnormal visual assessments or those who could not be assessed (F [2, 1032] = 1.97, *p* = 0.0001, ANOVA). There was a significant difference in mean discharge periods in comparison to level of Barthel score for general stroke disability impact (F [2, 1092] = 1.50, *p* = 0.0001, ANOVA). Those with abnormal visual assessments had greater severity of stroke as indicated by the Barthel score (*p* = 0.0001, Mann-Whitney test).

**Fig 4:**
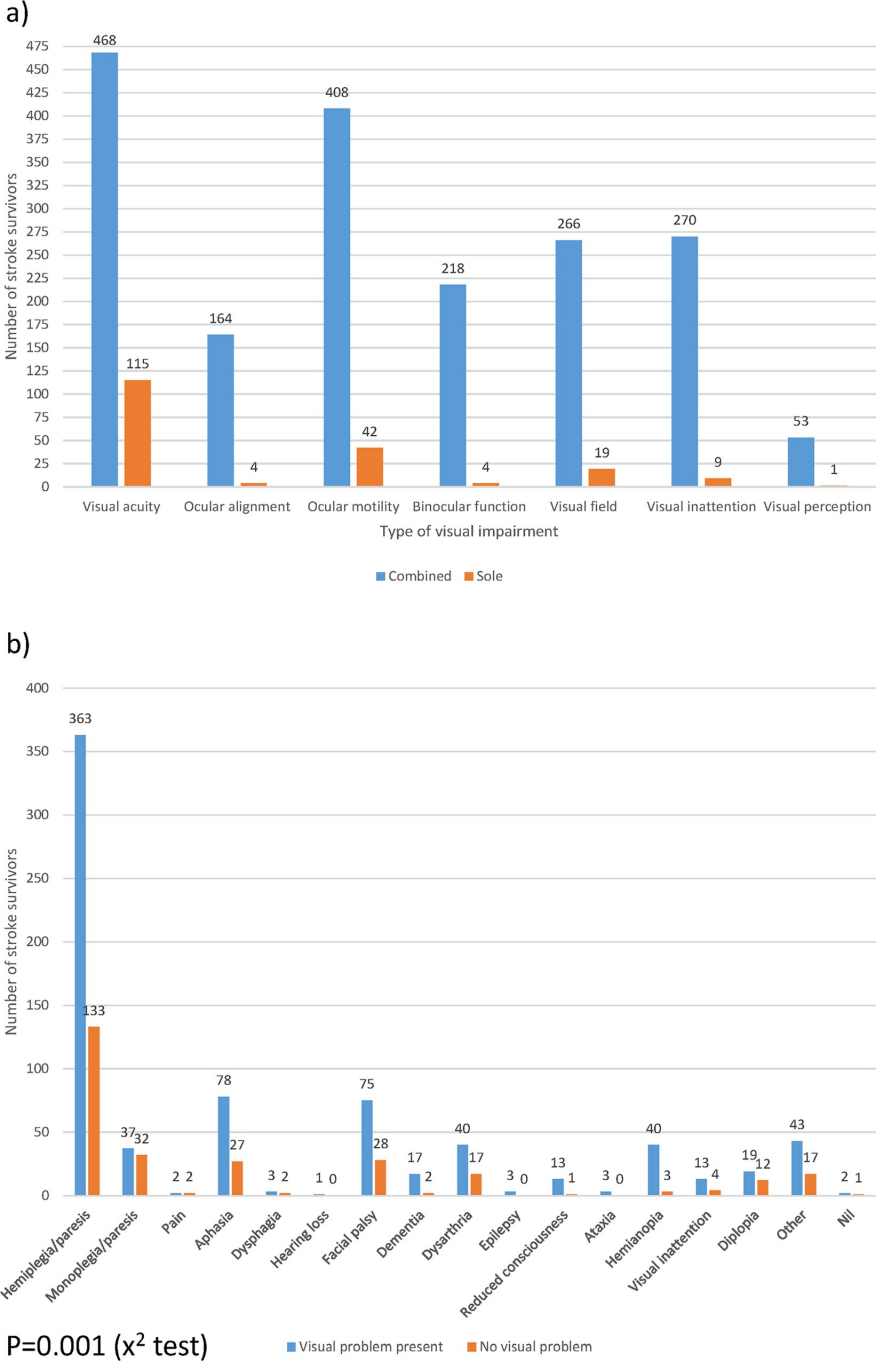
Categories of visual problems as sole or combined visual deficits and presence of visual problems versus primary systemic disability on admission. (a) 186 stroke survivors had a single issue with visual function as outlined in orange. Most (566 stroke survivors) had two or more visual problems–outlined in blue. (b) Stroke survivors most commonly had a hemi- or mono-plegia/paresis. The spread of primary general disability was similar for those who did or did not have a visual problem.

Abnormal findings included impaired central visual acuity (n = 583: 56.4%), ocular motility disorders (n = 519: 40.1% [inclusive of ocular misalignment (n = 168: 16.3%), ocular motility problems (n = 450: 43.6%), impaired binocular function (n = 222: 21.5%)], visual field loss (n = 285: 27.6%), visual inattention (n = 279: 27%) or visual perception (n = 54: 5.2%). Visual problems affected just one category in 186 stroke survivors (18%) or a combination of various categories in 566 stroke survivors (54.8%); see Fig 4A.

There was a significant difference in mean discharge periods in comparison to level of Barthel score for general stroke disability impact (F [2, 1092] = 1.50, p = 0.0001, ANOVA). Those with abnormal visual assessments had greater severity of stroke as indicated by the Barthel score (p = 0.0001, Mann-Whitney test). However, in terms of main systemic deficit, a range of issues were spread similarly for those with and those without a visual problem; see Fig 4B.

### Outcome of visual assessment–incidence

**Fig 5:**
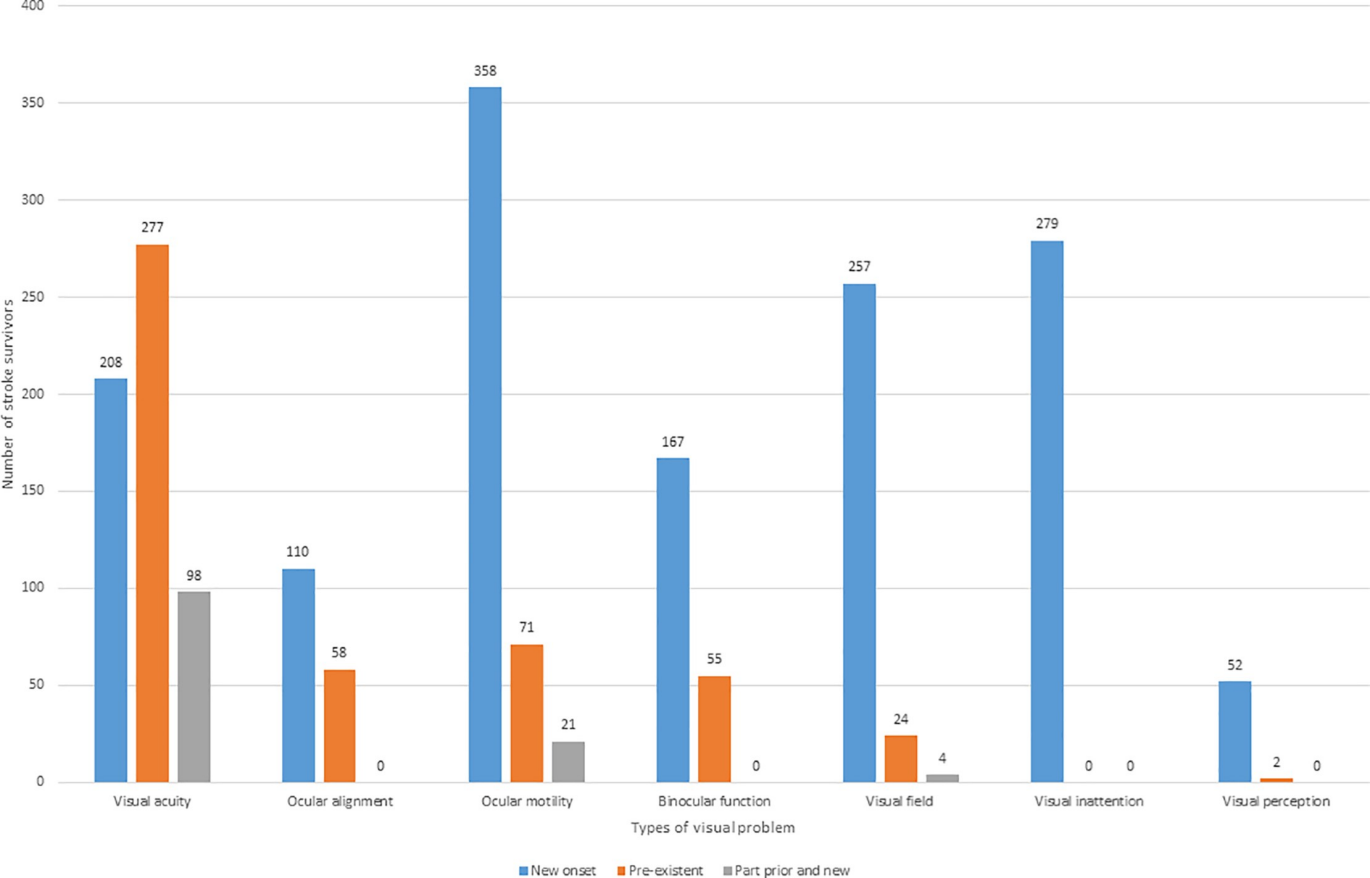
Categories of visual problems; pre-existent, part or new onset. Visual problems was categorised into new onset (332 stroke survivors–blue bars), pre-existent visual problems (136 stroke survivors–orange bars) and mixed new and pre-existent visual problems (284 stroke survivors–grey bars).

To evaluate incidence of new stroke-related visual sequelae, we made checks of hospital ophthalmic records and/or contact with the patient’s optician and/or patient recount of past ocular history and/or family/carer recount of past ocular history. We sought information about visual problems potentially associated with absent or incorrect glasses for refractive error, pre-existent ocular pathology, age-related eye movement disorders, pre-existent strabismus and/or amblyopia and longstanding visual symptoms. Most pre-existent visual problems occurred for visual acuity (277 of 582 cases) and the least for visual perception (2 of 54 cases). Overall, 136 stroke survivors had visual problems that pre-existed their stroke, 284 had a combination of pre-existent and new onset visual sequelae and 332 had new onset stroke-related visual sequelae (Fig 5). Thus, 616 stroke survivors (47.6% of stroke admissions, 59.6% of vision assessments) had abnormal findings on their visual assessments that were diagnosed as new incident cases following stroke. Incidence, and indeed point prevalence, varied across age groups which was proportional to the age of stroke onset and maximal in the age groups of 60–89 years (Table 3).

**Table 3:** Point prevalence and incidence figures across age groups.

	Age groups (years)								
	18–29	30–39	40–49	50–59	60–69	70–79	80–89	90–99	Totals
**Stroke admissions**	10 0.8%	8 0.6%	64 4.9%	127 9.8%	251 19.4%	334 25.8%	378 29.2%	123 9.5%	n = 1295 % of 1295
**Stroke assessments**	9 0.9%	4 0.4%	30 2.9%	64 6.2%	94 7.3%	128 12.4%	114 11.0%	27 2.6%	n = 1033 % of 1033
**Visual problems incidence**	3 0.2% 0.3%	3 0.2% 0.3%	29 2.2% 2.8%	50 3.9% 4.8%	107 8.3% 10.4%	146 11.3% 14.1%	187 14.4% 18.1%	68 5.3% 6.6%	n = 616 % of 1295 % of 1033
**Visual problems prevalence**	5 0.4% 0.5%	4 0.3% 0.4%	36 2.8% 3.5%	58 4.5% 5.6%	133 10.3% 12.9%	212 16.4% 20.5%	244 18.8% 23.6%	77 5.9% 7.5%	n = 752 % of 1295 % of 1033
**Impaired central vision** **n = 583**	3 0.2% 0.3%	2 0.15% 0.2%	19 1.5% 1.8%	38 2.9% 3.7%	88 6.8% 8.5%	171 13.2% 16.6%	191 14.7% 18.5%	71 5.5% 6.9%	n = 583 % of 1295 % of 1033
**Visual field loss** **n = 285**	0 0.0% 0.0%	1 0.1% 0.1%	14 1.1% 1.4%	21 1.6% 2.0%	54 4.2% 5.2%	78 6.0% 7.6%	90 6.9% 8.7%	27 2.1% 2.6%	n = 285 % of 1295 % of 1033
**Visual inattention** **n = 279**	1 0.1% 0.1%	1 0.1% 0.1%	9 0.7% 0.9%	18 1.4% 1.7%	47 3.6% 4.5%	78 6.0% 7.6%	88 6.8% 8.5%	33 2.5% 3.2%	n = 279 % of 1295 % of 1033
**Visual perceptual deficit** **n = 54**	0 0.0% 0.0%	1 0.1% 0.1%	0 0.0% 0.0%	5 0.4% 0.5%	17 1.3% 1.6%	16 1.2% 1.5%	12 0.9% 1.2%	3 0.2% 0.3%	n = 54 % of 1295 % of 1033
**Eye movement disorder** **n = 519**	4 0.3% 0.4%	4 0.3% 0.4%	26 2.0% 2.5%	38 2.9% 3.7%	93 7.2% 9.0%	143 11.0% 13.8%	149 11.5% 14.4%	49 3.8% 4.7%	n = 519 % of 1295 % of 1033

Table 4 outlines the duration from onset of stroke to the time at which a diagnosis of visual problems was made. Diagnosis of visual problems was made for nearly 90% of stroke survivors within the hyper-acute/acute (≤2 to 3–30 days of stroke onset). This was similar for all types of visual problems although slightly more cases of visual inattention were diagnosed within the hyper-acute and acute periods (96%). There were a number of stroke survivors who, despite attempts to offer visual assessment in the early acute stages post stroke, were finally assessed for vision at time points beyond 3–6 months. These stroke survivors were diagnosed with a variety of visual problems types, predominantly visual field loss and eye movement disorders which could be directly attributed to their stroke.

**Table 4:** Duration from onset of stroke to time of visual problems diagnosis.

	Hyper-acute	Acute				Sub-acute					Chronic
Days post stroke	≤2	3–7	8–14	15–21	22–30	31–61	62–91	92–122	123–152	153–182	≥183
**Visual problems incidence** **n = 616**	179	230	115	25	10	39	11	2	3	0	2
	179 (29%)	380 (61.7%)				55 (8.9%)					2 (0.3%)
**Visual problems prevalence** **n = 752**	216	276	138	28	14	54	14	4	4	1	3
	216 (28.7%)	456 (60.6%)				77 10.2%)					3 (0.4%)
**Impaired central vision** **n = 583**	168	224	109	21	12	36	6	3	1	0	3
	168 (28.8%)	366 (62.8%)				46 (7.9%)					3 (0.5%)
**Visual field loss** **n = 285**	76	101	59	14	6	19	6	1	2	0	1
	76 (26.7%)	180 (63.2%)				28 (9.8%)					1 (0.4%)
**Visual inattention** **n = 279**	82	108	61	13	4	8	3	0	0	0	0
	82 (29.4%)	186 (66.7%)				11 (3.9%)					0 (0.0%)
**Visual perceptual deficit** **n = 54**	14	19	10	2	3	3	1	0	1	0	1
	14 (25.9%)	34 (62.9%)				5 (9.4%)					1 (1.8%)
**Eye movement disorder** **n = 519**	134	186	111	23	9	38	13	2	0	1	2
	134 (25.8%)	329 (63.4%)				54 (10.4%)					2 (0.4%)

## Discussion

This is, to our knowledge, the first large study of stroke admissions over a 1-year period to meticulously determine the incidence of stroke-related visual sequelae and timing of vision assessment. The first attempted visual assessment was made at a median of 3 days post stroke onset. The time-point at which most stroke survivors could achieve a full visual assessment was at a median of 4 days post stroke onset. This contrasts with the Vision In Stroke (VIS) study which recruited stroke survivors referred for orthoptic assessment because of suspected visual problems at a median of 22 days (mean 40.8) [9]. However, by the nature of the VIS referral—because of suspected visual problems—this indicates prior screening in which stroke teams used a standardised screening form [10]. Thus, a later time-point to full assessment is to be expected from the VIS study.

The earlier assessment time-point for the IVIS study is important as it shows the feasibility and acceptability of early visual assessment within 3 days of stroke onset for at least half of stroke survivors and within 1 week of stroke onset for the majority. This in turn allows early detection of visual problems and sharing of the functional significance of this with the patients, carers and stroke teams. Furthermore, early assessment leads to early intervention, which has potential impact on general rehabilitation where visual function can be improved. Even for a very small subset of stroke survivors in our study who had late visual assessment (in the chronic phase of stroke) despite attempts to provide earlier vision assessment appointments, their visual problems could be directly attributed to their stroke and individualised treatment was commenced at those time points for these cases.

The three stroke units in the IVIS study were 24, 28 and 28 bed stroke units. The IVIS study allocated 0.1WTE (1 session per week) orthoptic staff per 10-bed unit as per national guidelines and achieved all required visual assessments, follow-up assessments and administration within the allotted staffing [8]. Thus, the national guidelines were validated as an appropriate staff-resourcing guide. Arguably, vision services should be organised based on the estimated sample of stroke survivors that can be assessed–in this study this was 1033 individuals (79.8% of stroke admissions). These are important considerations for commissioning and/or maintaining vision services on stroke units. Indeed the most recent 5^th^ edition of the UK National Clinical Guidelines for Stroke names orthoptists as part of the core acute multi-disciplinary stroke team [11].

For transparency, we report point prevalence and incidence of visual problems for all stroke admissions and for all stroke survivors who could be visually assessed. Where point prevalence and incidence is required for planning provision of vision services, the latter figures for point prevalence (72.8%) and for incidence (59.6%) are clinically relevant. When considering all stroke survivors, those who were never assessed (accounting for 172 of stroke admissions) should be reviewed. Many stroke survivors could not be assessed because they were unwell, had extreme fatigue or lacked sufficient cognition or attention to comply with vision assessment. It is widely acknowledged that strokes of greater severity are likely to cause visual disturbance [12]. Thus, we suggest it is likely that a proportion of these stroke survivors had visual problems of some form.

Discharge from the stroke unit for all stroke survivors was made at a mean of 38 days with those who had normal vision assessments having significantly earlier mean discharges than those who had visual problems confirmed (13.5 versus 50 days). We found that those with abnormal visual assessment, had greater stroke severity indicated by low Barthel scores. However, in terms of presence or absence of systemic deficits, there was a similar spread of such deficits in those that did have visual problems as well as those that did not.

Some stroke survivors could not be assessed because they were discharged from hospital prior to visual assessment being undertaken. For those discharged early (within 2 days of stroke onset), many individuals had less severe strokes as indicated by higher Barthel scores, and had recovered well enough to allow for early discharge [13]. However, it cannot be assumed that they had no visual problems. Previous research in the VIS study showed 16% of stroke survivors to be asymptomatic despite presence of significant visual problems [9]. For the IVIS study stroke survivors who were discharged later, this was often to care homes because of stroke severity and lasting disability. These individuals are also at more risk of having visual sequelae [12]. Thus, we consider that 60% incidence of visual problems for all stroke survivors should be considered as an absolute minimum and potentially represents an under-estimate based on the unknown visual status of these unassessed stroke survivors.

The point prevalence or incidence of visual problems varied by age but was proportional to the age at onset of stroke, i.e. increasing rates of visual problems with increasing age. Most stroke onsets were from age 60 to 89 years and most cases of visual problems were also seen in these age groups. There are few UK-based population studies of adult visual problems so it is not possible to directly make comparisons of our results. However, general similarities and dissimilarities can be considered. A national UK review reported point prevalence of visual problems with impaired central vision of less than 0.3 logMAR (as in our study) at 13–19% for persons aged 65–74 years, 16–33% for those aged 75–84 years and 42–61% for those aged 85 years and older [14]. Visual problems in this UK review could be due to any cause and was typically due to eye disease. Strabismus is reported to occur in about 4% of the population [15,16]. One recent US study similarly reported a life-time risk of adult-onset strabismus at 4% but also noted a significant increase in incidence of strabismus with increasing age showing peak incidence in the eight decade of life [17]. Our figures show similar increases with age, with impaired central vision prevalence peaking at 18.5% and eye movement disorders peaking at 14.4% for ages 80–89 years. However, we report higher prevalence rates than those reported in the literature for the general population when considering the underlying cause is stroke alone.

The breakdown of type of visual problems showed the greatest percentage to have impaired central vision followed by eye movement disorders, visual field loss and visual perceptual disorders. Some visual problems occurred as a single visual problem but most individuals had a combination of visual problems. Visual field loss, specifically homonymous hemianopia, is recognised as a common visual consequence of stroke but was not as common as impaired central vision or eye movement disorders. Impaired central vision is well reported however, as are eye movement disorders [18–21]. Visual field loss occurs following damage to the visual pathway, most frequently in the retro-chiasmal pathway [6] whilst strabismus and eye movement disorders occur due to direct damage to the cortical, nuclear or infranuclear pathways that generate eye movements [20,22].

Impaired central vision occurring in stroke survivors is often due to coexistent ocular problems [18]. Over half of causes of visual problems in persons aged 75 years upwards can be attributed to needing glasses or cataract [14]. Given the strong likelihood of impaired central vision being related to causes other than stroke, we specifically sought verification of ocular history within the year prior to stroke onset and, further, where new onset impaired central vision was noted, we made added checks for refractive error and ophthalmic pathology as part of the full specialist vision assessments to enable accurate diagnosis. We accounted for cases where reduced central vision could be attributed to the need for glasses update or new prescription, or to ocular conditions such as cataract, age-related macular degeneration, glaucoma or other eye disease plus for childhood onset conditions such as amblyopia and strabismus. Remaining cases with impaired central vision following stroke may be postulated to occur because of stroke-related impact to the visual pathway. Visual symptoms are common in cerebrovascular disease with one example being amaurosis fugax where temporary symptoms of dimming or loss of vision is reported. Good central vision requires healthy vascular perfusion of the retina with arterial blood supplied by the central retinal artery (branch of the anterior brain arterial circulation). Following stroke symptoms of blurred vision can persist and may reflect an effect of reduced arterial circulation and relative ischaemic hypoxia in the anterior visual pathway which, for many stroke survivors, improves during subsequent weeks [3].

Symptoms related to these visual problems include blurred or altered vision and loss of visual field and awareness [7]. These symptoms cause considerable impact to quality of life resulting in loss of independence, loss of confidence, social isolation, impaired mobility and depression [23–25]. Thus, these are important problems to detect and diagnose early so that early and prompt interventions can be targeted for the individual. There are a variety of interventions that can be individually tailored to address the visual symptoms and consequences of stroke-induced visual sequelae. For example, an eye patch or monocular prism are effective in blocking or correcting double vision due to eye movement disorders [26,27]. Compensatory visual scanning and search training shows benefit for stroke survivors when adapting to hemianopic visual field loss [26, 28]. A number of systematic reviews are available that discuss these interventions [26–29].

### Limitations

The main limitation to this study is that the stroke population was confined to the North West of England with recruitment from three stroke units. However, the population demographics for our study are similar to the reported demographics for the North West of England [30], and we suggest that our results might be cautiously generalisable to other UK areas with stroke populations. Further study of incidence and prevalence of visual problems in other areas with greater numbers of non-Caucasian ethnicities or younger stroke survivors might also be warranted.

There are over 100,000 new strokes per annum in the UK [31]. In light of our high incidence of stroke-related visual sequelae, a worrying large number of stroke survivors may not be getting rapid competent vision assessments [32, 33]. This may risk long-term harms in terms of their well-being, function, mobility and independence [23], and hence avoidable excessive costs to the NHS with wider implications such as costs to families and social care.

Our results are potentially of relevance to other countries outside the UK. Considering the high prevalence and incidence of visual problems from our prospective acute stroke unit study, it is likely that other acute stroke unit populations would have high proportions of stroke survivors with visual problems. Challenges arise with regard to how best to visually screen stroke survivors. Many countries have access to orthoptic services but, in the absence of such services, involvement of other eye care professionals (e.g. ophthalmologists and optometrists) should be considered. In addition, implementation of standardised vision screening with validated screening tools administered by members of the stroke team, can also improve the detection of visual problems in stroke survivors [34].

## Conclusions

In this population of acute stroke unit admissions, point prevalence of visual problems ranges from 58% for all stroke admissions to 73% for all stroke survivors undertaking visual assessment. Incidence of new onset visual sequelae due to stroke ranges from 48% for all stroke admissions to 60% for all stroke survivors undertaking visual assessment.

Although a research study, this study of acute stroke admissions was planned and undertaken in accordance with national clinical guidelines for staffing allocation and using standardised clinical vision assessments. Early visual screening and assessment is feasible and achievable within 72 hours of stroke onset and for those initially unable to be visually assessed, most can be assessed within one week of stroke onset. It is important to detect visual problems, regardless of whether it is pre-existent or of new onset, and disseminate the functional consequences and impact of this to patients, carers and stroke teams so this can be accounted for in activities of daily living and general mobilisation/rehabilitation. There are likely wide-ranging benefits to patients, their carers and the NHS through early and accurate identification of stroke-related visual problems.

## Supporting information

S1 File IVIS database.sav Minimum anonymised data set. (SAV)
